# Animal Models for the Study of Keratoconus

**DOI:** 10.3390/cells12232681

**Published:** 2023-11-22

**Authors:** Rachel Hadvina, Amy Estes, Yutao Liu

**Affiliations:** 1 Department of Cellular Biology & Anatomy, Augusta University, 1120 15th Street, Augusta, GA 30912, USA; 2 Center for Biotechnology and Genomic Medicine, Augusta University, 1120 15th Street, Augusta, GA 30912, USA; 3 James & Jean Culver Vision Discovery Institute, Medical College of Georgia, Augusta University, 1120 15th Street, Augusta, GA 30912, USA; 4 Department of Ophthalmology, Augusta University, Augusta, GA 30912, USA

**Keywords:** keratoconus, cornea, animal models, mice, rabbit, keratocytes

## Abstract

Keratoconus (KC) is characterized by localized, central thinning and cone-like protrusion of the cornea. Its precise etiology remains undetermined, although both genetic and environmental factors are known to contribute to disease susceptibility. Due to KC’s complex nature, there is currently no ideal animal model to represent both the corneal phenotype and underlying pathophysiology. Attempts to establish a KC model have involved mice, rats, and rabbits, with some additional novel animals suggested. Genetic animal models have only been attempted in mice. Similarly, spontaneously occurring animal models for KC have only been discovered in mice. Models generated using chemical or environmental treatments have been attempted in mice, rats, and rabbits. Among several methods used to induce KC in animals, ultraviolet radiation exposure and treatment with collagenase are some of the most prevalent. There is a clear need for an experimental model animal to elucidate the underlying mechanisms behind the development and progression of keratoconus. An appropriate animal model could also aid in the development of treatments to slow or arrest the disorder.

## 1. Introduction

Keratoconus (KC) is a bilateral, asymmetric corneal disorder characterized by localized central thinning and cone-like protrusion of the cornea. Symptoms of KC begin to develop during puberty and young adulthood [1]. The human cornea is divided into five distinct layers and is responsible for protecting ocular structures, the refractive power of the eye, and focusing light on the retina [2]. A progressive and degradative disorder, KC presents as irregular astigmatism, myopia, and corneal scarring, which worsen over time and can result in vision loss [3]. At the tissue level, KC is marked by centralized thinning of the stroma, breaks in Bowman’s layer, compaction of the collagen fibers in the stroma, and iron deposition in the epithelial basement membrane [1,4,5,6,7]. All five layers of the cornea may be affected by KC pathogenesis, but the characteristics mentioned are well-recognized markers of KC [1]. In addition, the epithelium may exhibit degeneration and breaks, and the fibril arrangement of the stroma may be altered, among other changes to corneal structures [1]. Clinically, KC is diagnosed by corneal topography [8]. Corneal topography confirms the abnormal corneal curvature characteristic of keratoconus, but other morphological defects may be used to confirm a diagnosis [8]. The prevalence of KC globally ranges between 0.2 and 4790 per 100,000 people [8]. The prevalence of KC in the United States is often reported based on a 1986 study as 54.5 per 100,000 people [9]. More recent estimates vary, but there is no well-established current prevalence rate for KC in the United States. The management of symptoms ranges from minimally invasive measures like rigid contact lenses to more invasive collagen crosslinking (CXL) and surgical interventions such as total corneal transplant or refractive surgery [8].

The exact etiology of KC is undetermined, although genetic and environmental factors are known to contribute to disease susceptibility [10]. Family history is known as a risk factor for KC, substantiating the notion that genetic factors contribute to KC pathogenesis [11]. The prevalence of KC is notably higher between affected individuals and first-degree relatives than in the general population [12]. However, many cases of KC are sporadic and follow no evident inheritance pattern [13]. Genome-wide association studies (GWAS) and other genomic studies have been conducted to determine which genes may be associated with KC [14]. Some genes identified in these GWAS include *COL5A1*, *HGF*, *ZNF469*, and *MPDZ/NF1B* [15,16,17,18,19,20]. Additionally, the involvement of biological pathways, including wound healing, collagen structure and synthesis, cell growth and proliferation, and proteolytic degradation, was identified through genomic analyses [14]. Gene–environment interactions are also believed to contribute to the complex nature of the disorder. Some environmental and behavioral risk factors include eye rubbing [21], ultraviolet radiation (UVR) exposure [22], and atopy [11]. KC susceptibility is also associated with other ocular and systemic syndromes, such as anterior polar cataracts [23], Down’s syndrome [24], Ehlers–Danlos syndrome [25], and others.

Due to the complexity of keratoconus, there is currently no established animal model to represent the corneal phenotype and underlying pathophysiology. An appropriate animal model for KC would demonstrate a cone-like morphology, localized thinning of the central cornea, and degradation of corneal layers. In regard to the corneal layers specifically, we expect to see the previously mentioned histological characteristics, including stromal and epithelial thinning, the compaction of stromal collagen fibers, and epithelial iron deposition. Depending on the species, we would expect to see breaks in the Bowman’s layer; however, some animals lack this layer in the cornea. In order to characterize these phenotypes, various experimental techniques may be used, including optical coherence tomography (OCT), slit lamp biomicroscopy, corneal topography mapping, scanning electron microscopy, transmission electron microscopy, and histology. Unfortunately, not all of these techniques are available for each model animal.

The lack of an accurate animal model for KC limits research to clinical studies, ex vivo human donor corneas, and potentially insufficient animal models. Attempts at developing a model to represent KC have involved mice, rats, rabbits, and some human tissue culture and both 2D and 3D in vitro human cell culture methods. Some other non-traditional animal models have been suggested to study corneal ectasia, but none have been employed for this purpose. There is a need for a robust experimental model animal to help to reveal the underlying mechanisms of KC development and progression, as well as to discover new treatments. In this review, we will discuss the current landscape of non-human animal models in KC research, as summarized in Table 1, and review suggestions and future directions to develop an accurate laboratory model for keratoconus.

## 2. Mouse Models

The ease of handling, housing, and breeding of mice contributes to their wide use in research, including in ocular research. The ample resources available for the genetic modification of mice make them very useful in functional studies. The mouse eye is drastically smaller than the human eye and is smaller than that of other laboratory animals (Table 2) [51,52]. Some strains of mice have five distinct corneal layers; however, the presence of a Bowman’s layer is disputed [53,54]. One important difference between the mouse and human cornea is the variation in thickness in the mouse stroma. Mouse corneas vary in stromal thickness across the diameter, with the center being slightly thicker than the periphery [55]. This is relevant as an appropriate mouse model for KC will demonstrate thinning of the central cornea. The mouse cornea exhibits an average central corneal thickness (CCT) of 106.0 µm, whereas the human cornea shows a CCT of 565.0 µm (Table 2) [56,57]. The mouse cornea is fully developed after 8 weeks [58].

Certain environmental influences and chemical treatments such as UVR exposure, atopic dermatitis, collagenase, and endo-β-galactosidase have been used to induce a KC-like phenotype in mice [26,27,28,29]. Some spontaneously occurring KC-like murine lines have also been noted [30,31]. Additionally, genetic mouse models based on genes of interest to KC have been attempted [33,34,35,36,37,38,39,71]. However, genetic models may not accurately capture cases of KC influenced by environmental factors or other risk factors. Without a complete understanding of the genetic etiology of KC in humans, it is difficult to confirm that the driving factors in genetic models are the same ones driving hereditary KC in humans.

### 2.1. Treatment-Induced Mouse Models

**Figure 1:**
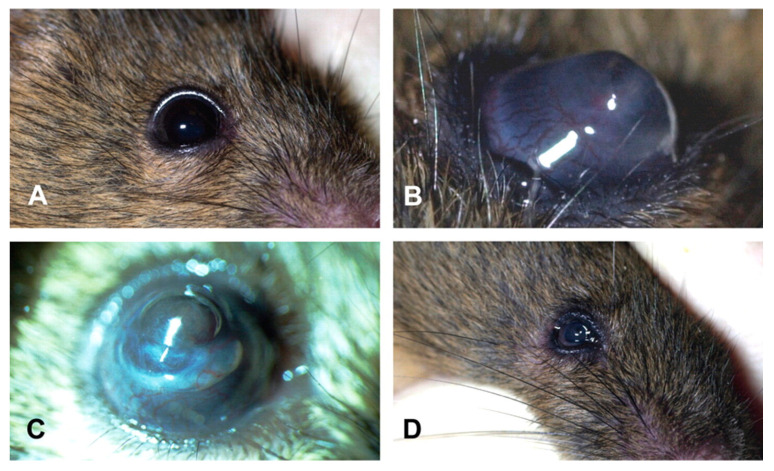
129S1/SvImJ mouse eyes in the (**A**) normal and (**B**) keratoconic conditions. (**C**) Corneal perforation and iris prolapse. (**D**) Phthisis bulbi. (Used with permission of SAGE Publications Thousand Oaks, CA, USA, from Ultraviolet Radiation-Induced Corneal Degeneration in 129 Mice, Newkirk et al., TOXICOLOGIC PATHOLOGY, Volume 35, Issue 6, p. 817–824, 2007, https://doi.org/10.1080/01926230701584197, accessed 29 September 2023) [26].

One environmental influence contributing to the development and progression of KC is exposure to ultraviolet radiation (UVR) [22]. Newkirk et al. observed corneal protrusion and degeneration consistent with KC as a byproduct of UVR exposure during a skin carcinogenesis study [26]. In the study, 129S1/SvImJ mice were exposed to a mixture of 40% UVA and 60% UVB at a dose of 3200 J/m^2^, which resulted in corneal abnormalities such as lesions, stromal cleft formation, stromal thinning, corneal vascularization, fibrosis, inflammation, and perforation (Figure 1) [26]. Molecular changes to the stroma included hypo-cellularity and collagenolysis [26]. Epithelial hyperplasia and dysplasia were likewise observed [26]. Many of the observations made in this study indicate severe corneal injury. Still, stromal thinning and cell loss are consistent with the central corneal thinning characteristic of KC. One drawback of this mouse model is the discomfort of the animals caused by exposure to UVR, which is apparent from the observed skin and corneal damage. It may be possible to remedy ethical concerns and better control ectasia development by adapting this method to use a lower dose of UVR over a longer duration. An adaptation to the strength of the UVR, the frequency of treatment, and/or the length of treatment may lessen the level of damage to the skin and eyes while still producing the necessary phenotype.

As previously mentioned, atopy and eye rubbing are risk factors for KC [11,21]. Ebihara et al. studied the effects of atopic dermatitis, an allergic condition of the skin, on corneal defects [27]. Since atopic dermatitis manifests as an intense scratching behavior in NC/Nga mice, this study observed changes to the corneal morphology and structure as a direct result of eye rubbing [27]. In this study, eye rubbing caused thinning of the epithelium; an irregular interface between the epithelium and stroma; the invasion of fibrotic keratocytes in the epithelium; hemidesmosome accumulation in the basal processes of basal cells; the deposition of material under epithelial cells; the deformity of keratocytes and disorganization of collagen fibers in the stroma; and neovascularization of the stroma [27]. Importantly, these mice acquired cone-shaped corneas [27]. A significant quantity of apoptotic cells were identified in the epithelium, consistent with cell damage to this layer [27]. The application of this method as a mouse model for keratoconus may not be practical at a large scale due to ethical and animal use concerns. It is also difficult to determine whether the atopy contributes to the corneal phenotype or whether the eye rubbing alone, as a consequence of atopy, is the primary contributor. However, since eye rubbing is a well-recognized contributor to KC, it is a promising possibility.

In order to induce KC in mice, Moghadam et al. utilized injections of collagenase directly into the corneas of mice [28]. Collagenase is an enzyme that breaks down collagen proteins and is implied in KC-associated stromal thinning [72]. One of the main treatments for keratoconus is collagen crosslinking, which repairs chemical bonds between collagen fibrils [73]. In essence, this experiment sought to achieve the direct opposite, breaking the bonds between collagen fibrils in the cornea to induce the structural changes present in KC. This study found that a specific dose of collagenase in the corneas of male mice induced damage to collagen fibrils, thinning of the cornea and epithelium, and corneal rupture [28]. Unfortunately, only male mice were used in this study, despite the lack of a sex-dependent phenotype. It would be very helpful for this experiment to be replicated using a population of mice without any bias for sex and to perform diagnostic measurements. Collagenase has also been used to induce KC in rat and rabbit models, which will be discussed in later sections, making it a promising possibility.

Another treatment proposed to induce KC in mice is endo-β-galactosidase. Endo-β-galactosidase is a proteoglycan responsible for the hydrolysis of keratan-sulfates [74]. Keratan-sulfates are glycosaminoglycans found in the cornea; proteoglycans in general are believed to contribute to the stromal structure [32,75]. Bech et al. described the use of endo-β-galactosidase to induce KC in mice by degrading the proteoglycan extracellular matrix (ECM) [29]. Keratectomy of the epithelium followed by treatment with endo-β-galactosidase was performed [29]. As a result, some corneas developed a cone-like shape, epithelial thinning, and stromal thinning [29]. A full-length publication based on the information presented in this abstract is not available to validate the results described. Still, the role of the ECM in the corneal stroma is well established, and endo-β-galactosidase as a means of disrupting the ECM structure in the cornea is a feasible option to mimic KC.

### 2.2. Spontaneous Mouse Models

**Figure 2:**
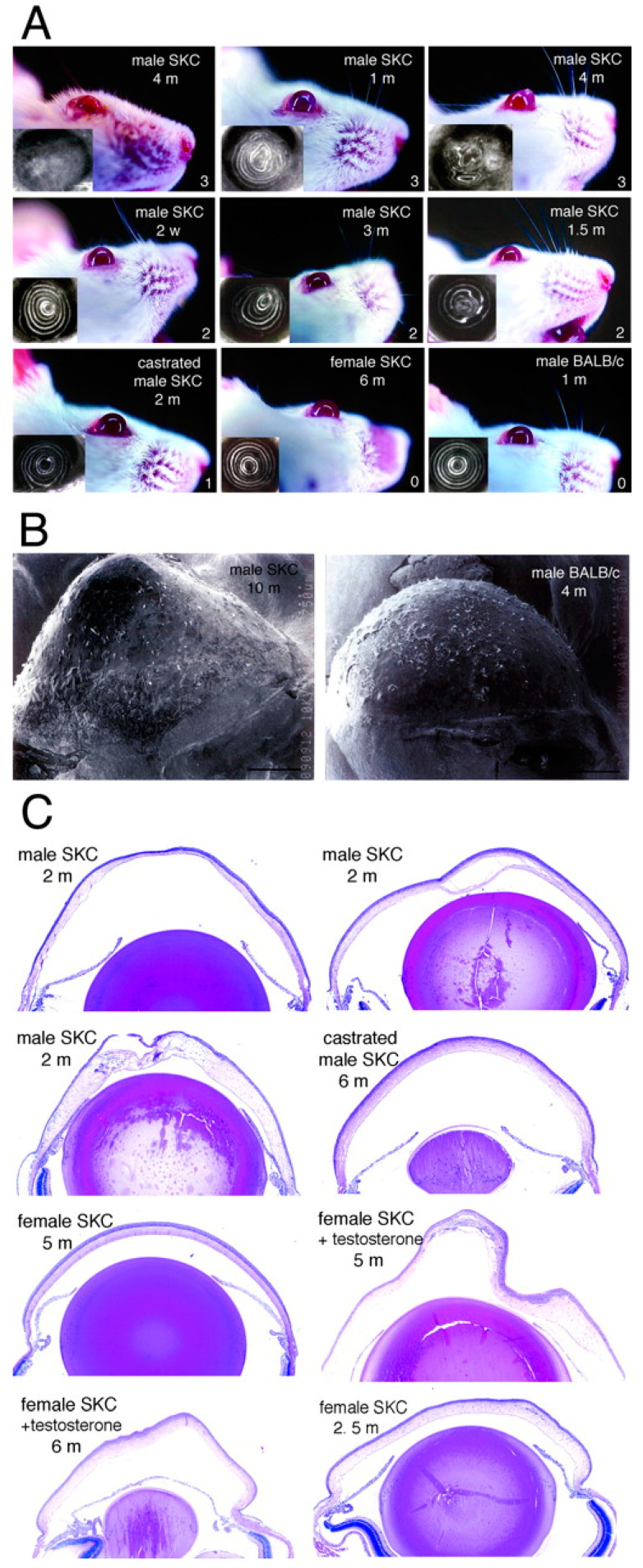
Keratoconus in SKC males and testosterone-injected SKC females. (**A**) Macroscopic and keratosocopic appearance of keratoconic SKC corneas. (**B**) Scanning microscopic appearance of SKC corneas. (**C**) Histologic appearance of SKC corneas. Original magnification, ×100. (Used with permission of Association for Research in Vision & Ophthalmology (ARVO) from Androgen-Dependent Hereditary Mouse Keratoconus: Linkage to an MHC Region, Tachibana et al., Volume 43, Issue 1, 2002]; permission conveyed through Copyright Clearance Center, Inc., Danvers, MA, USA) [31].

An interesting study from Tachibana et al. recorded a spontaneously occurring strain of mice with hereditary cases of KC [30]. This inbred line of mutant, wild Japanese mice (SKC) exhibited a KC phenotype with cone-shaped corneas (Figure 2) [31]. Corneal morphology, cell growth, apoptosis, and expression of c-fos, a transcriptional regulator previously associated with human KC, were assessed in the SKC mice (Figure 2) [30,31,76]. Affected mice exhibited inflammatory changes inconsistent with human keratoconus [30]. It was determined that the phenotype was passed between generations in an autosomal recessive manner with a bias for males [31]. Additionally, an androgen-dependent phenotype was discovered in these mice, with females developing KC when injected with androgen (Figure 2) [31]. In a follow-up study by Quantock et al., the structure of collagen fibrils in SKC mice was determined to be larger and more widely spaced than in a non-KC mouse line [77]. A spontaneously occurring line of KC mice could be an ideal model to mimic the underlying pathology of hereditary KC.

### 2.3. Genetic Mouse Models

A mouse model of trisomy 16 mice, developed by Tost et al., was originally used to model Down’s syndrome but noted ocular abnormalities in fetal mice [33]. The abnormalities identified included corneal hypoplasia, stromal fibrosis, the degradation of lens fibers, and the loss of compaction in stromal lamellae [33]. These abnormalities affected both the corneal epithelium and overall cornea morphology [33]. The defects were confirmed using hematoxylin and eosin (H&E) staining, Heidenhain’s AZAN trichrome staining, and periodic acid–Schiff staining [33]. It was speculated in the study that the mice may have developed keratoconus had they survived; however, without conclusive evidence, it is difficult to confirm this hypothesis [33]. Down’s syndrome is a well-established comorbidity to keratoconus that may have contributed to the observations made in this study [24]. This study provides a valuable and promising mouse model to study KC and Down’s syndrome together. However, this model is likely not suitable to study keratoconus without Down’s syndrome as a comorbidity. Additionally, all experimental assessments were made via histology. Without any measurements taken in live, fully mature mice, it is difficult to properly assert that keratoconus was developing.

**Figure 3:**
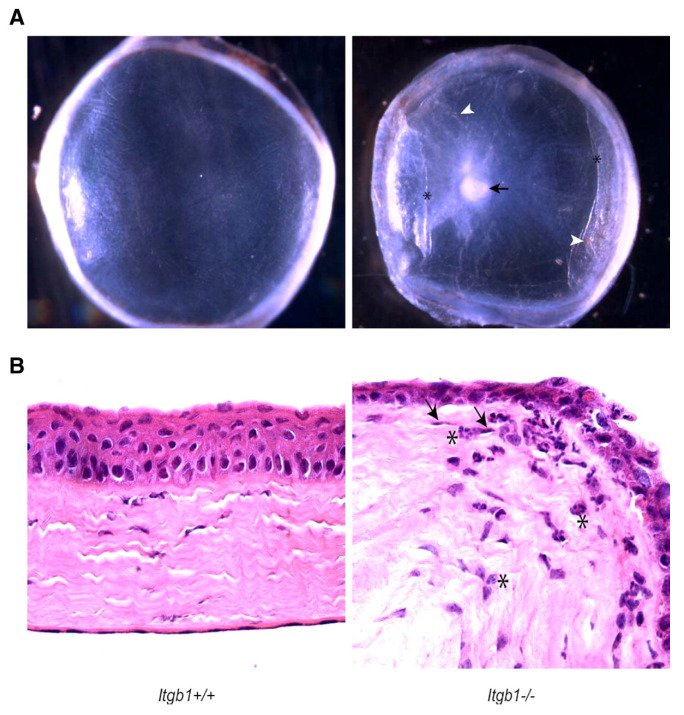
Morphology of Itgb1−/− corneas. (**A**) Uneven epithelial surface, scar tissue (arrow), corneal haze, and neovascularization. (**B**) Severe loss of epithelial layers, presence of neutrophils (asterisk), and endothelial cells around capillary lumen (arrows). (Used with permission of Association for Research in Vision & Ophthalmology (ARVO) from Integrin β1 Is Necessary for the Maintenance of Corneal Structural Integrity, Parapuram et al., Volume 53, Issue 11, 2011]; permission conveyed through Copyright Clearance Center, Inc. Danvers, MA, USA) [34].

Integrins are involved in the interactions between cells and their extracellular matrix and are implicated in the corneal structure [78]. Parapuram et al. conditionally deleted Itgb1 in a murine model via a tamoxifen-dependent Cre recombinase [34]. As a result of the Itgb1 deletion, the corneas exhibited stromal thinning, loss of epithelial cell layers, edema, scarring, and stromal haze [34]. The effects of the Itgb1 deletion were confirmed with H&E staining, immunofluorescence microscopy, and transmission electron microscopy [34]. It was clear that the conditional deletion conferred degenerative effects on the cornea, with the authors noting that this characteristic resembled KC (Figure 3) [34]. Still, there were traits inconsistent with the KC pathology, namely the edema and endothelial degeneration [34]. Importantly, when the knockout was induced after corneal maturation, none of the structural deficits arose [34]. Given the role of Itgb1 in the stromal extracellular matrix structure, this model may represent part of the complex genetic etiology of KC [78]. However, measurements in live mice would be beneficial to confirm the KC-like changes to the cornea. Likewise, certain diagnostic measurements like CCT and corneal topography are crucial to validate KC.

**Figure 4:**
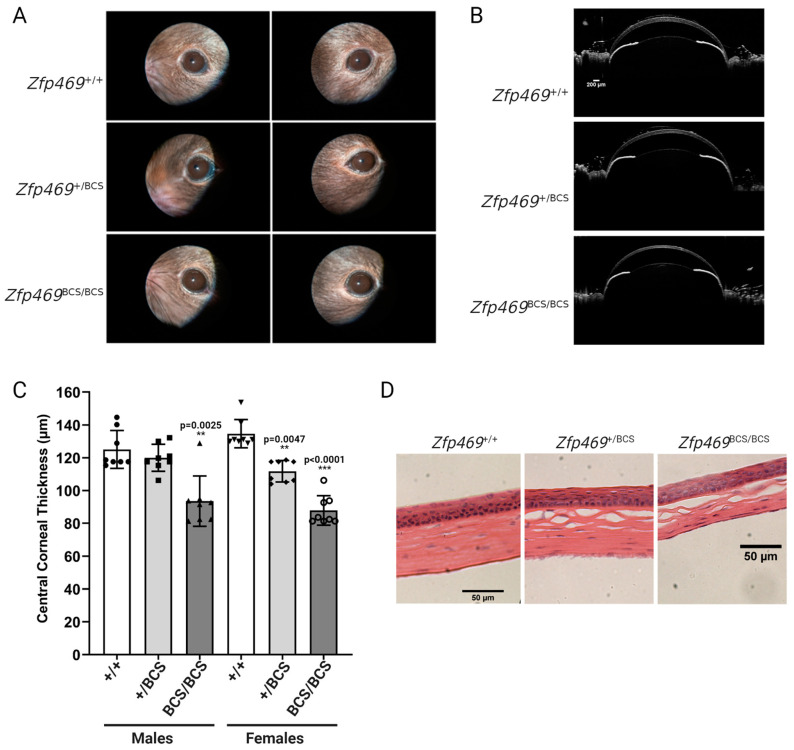
Corneal thinning in Zfp469BCS/BCS mice caused by stromal thinning. (**A**) Corneal opacity of mice examined by slit lamp. (**B**) Anterior segment OCT of Zfp469BCS/BCS, Zfp469+/+, and Zfp469+/BCS mice. Scale bars = 200 µm. (**C**) Reduced CCT in Zfp469BCS/BCS mice. (**D**) H&E staining of thinned corneal sections from Zfp469BCS/BCS mice. (Adopted from Stanton et al., https://creativecommons.org/licenses/by/4.0, accessed 14 November 2023) [35].

A brittle cornea mouse model developed by Stanton et al., with a homozygous mutation in the *Zfp469* (also referred to as *Znf469*) gene, demonstrated effects on the stroma and resulted in decreased collagen type I/collagen type V ratio expression [35]. *Zfp469* is one of two genes in which loss-of-function mutations confer brittle cornea syndrome, which is characterized by thinning of the cornea and sclera [35]. The mice in this study did not demonstrate the cone-like malformation of the cornea; however, some of the histological changes did resemble those seen in KC-affected corneas [35]. Of interest is the stromal thinning, which is consistent with the histopathology of KC (Figure 4D) [35]. Some shearing of the anterior portion of the stroma was also visible through H&E staining (Figure 4D) [35]. Crucially, the thinning was not progressive or localized as is seen in KC [35]. It is probable that the similarities in the histopathology of brittle cornea syndrome and keratoconus could be due to the role of *Znf469* in maintaining the corneal structure and thickness. In fact, *Znf469* been shown to regulate corneal ECM synthesis in a zebrafish BCS model [79]. Additionally, *Znf469* has been identified as a gene of interest in KC through GWAS [18,19]. This information provides sufficient rationale for further exploration of a *Zfp469* mouse model for KC.

**Figure 5:**
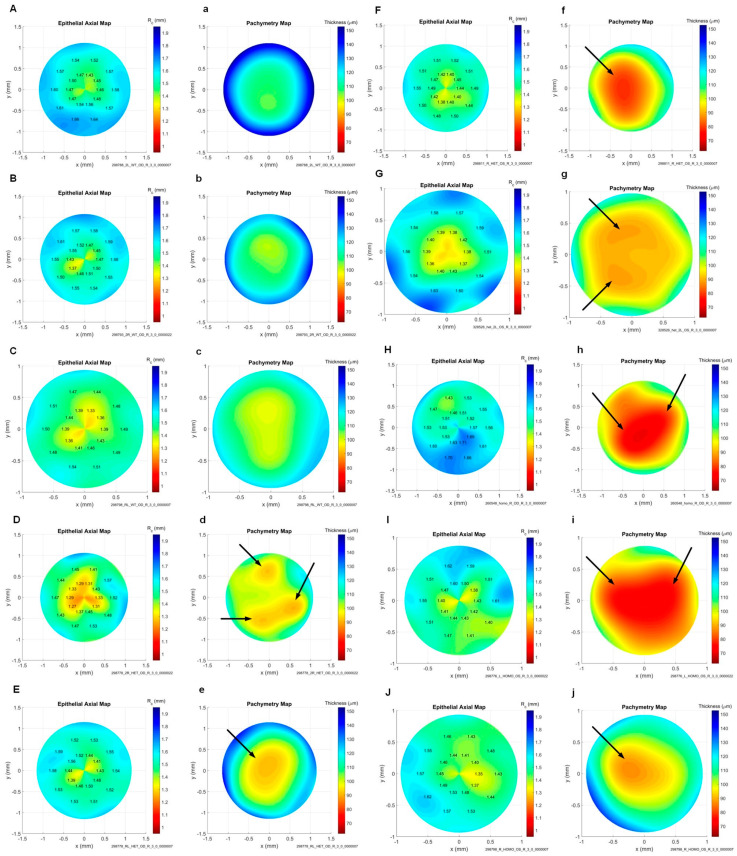
Corneal curvature and thickness mapping. Panels (**A**–**C**), (**a**–**c**) for wild-type *Ppip5k2*^+/+^ mice; (**D**–**G**), (**d**–**g**) for *Ppip5k2*^+/*K^*^ mice; and (**H**–**J**), (**h**–**j**) for *Ppip5k2^K^/K^^* mice. Abnormally thin regions are marked with arrows. (Adopted from Khaled et al., https://creativecommons.org/licenses/by/4.0, accessed 14 November 2023) [36].

A genetic mouse model of keratoconus developed by Khaled et al. was based on whole-exome and whole-genome sequencing from patients with hereditary KC [36]. Sequencing results from multi-generational families with KC showed that the *Ppip5k2* gene contained a non-synonymous mutation that could contribute to KC [36]. *Ppip5k2*, along with its homolog, is responsible for encoding functional enzymes with kinase and phosphatase domains [36]. A *Ppip5k2*-gene-trap mouse was used to replicate the increased levels of kinase activity and reduced levels of phosphatase activity shown in the ocular cells and tissues of patients with KC [36]. Some mice demonstrated abnormal corneal surfaces, changes in anterior chamber depth, thickened epithelia, abnormal corneal curvature, and thinning of CCT [36]. These traits were distributed among homozygous PPIP5K2 mutant and heterozygous mice and were not concurrently present in any given mouse [36]. The phenotypes of the gene-trap mice were validated using OCT, H&E staining, slit-lamp biomicroscopy, and pachymetry mapping (Figure 5) [36]. One of the major confounders in this model is the variability in phenotypes among mice of the same genotypes. Although this variability likely does represent the variability of KC in humans, it creates a challenge to control in a laboratory setting. However, the combination of methods employed to assess corneal phenotypes is an excellent roadmap for future studies to follow.

Terciero et al. described a prolactin-inducible protein (PIP) knockout developed with Clustered Regularly Interspaced Short Palindromic Repeats (CRISPR)/Cas9-mediated genetic modification [37]. PIP is a secretory acinar protein, identified as a potential biomarker in human tear fluid, that may differentiate between unaffected and keratoconic eyes [80]. Thus, a knockout model targeting this protein could help to understand how PIP affects the corneal structure in the context of KC. However, this study does not mention any phenotype exhibited by the knockout mice that is indicative of KC or any other disease. An appropriate model for KC will show clinical indicators and molecular changes to the corneal structure; neither were validated in this initial report. Until further assessments of the corneas are made, it is unclear what potential this model has aside from the suggested role of PIP in the corneal structure.

In their 2023 publication, Wang et al. proposed a mouse model of corneal ectasia involving a transforming growth factor beta receptor 2 (Tgfbr2) conditional knockout model [38]. This model is interesting as TGFB1 has previously been associated with KC through sequencing analysis [81]. The novel mouse strain, *Tgfbr2^kera-cko^*, was assessed for clinical indicators of corneal ectasia using OCT, slit-lamp biomicroscopy, and intraocular pressure (IOP) measurements [38]. Tissue and cell morphology were assessed using H&E staining, immunofluorescence microscopy, and transmission electron microscopy [38]. Eye rubbing was applied to the eyes in order to exemplify the corneal deformation seen in corneal ectasia patients [38]. These experiments revealed stromal-specific thinning in the *Tgfbr2^kera-cko^* mice, with a thickened epithelium, reduced TGFB2 expression in keratocytes, Col1a1 expression reduction, and diminished stromal collagen fibril density [38]. All of these results together provide a convincing model for corneal ectasia in mice that mimics the structural deficits and histopathological characteristics seen in humans. It is important to note that there was no localized corneal ectasia or thinning. As a consequence, these transgenic mice did not develop the characteristic cone-like morphology seen in KC. Furthermore, the study does not make mention of any gross, observable characteristics of the *Tgfbr2^kera-cko^* corneas to validate a cone-like morphology, although the authors do hypothesize that the abnormal shape may be a product of increased epithelial cell proliferation [38]. As an in vivo model of corneal ectasia alone, this study is sufficient and convincing. However, it does not seem that the *Tgfbr2^kera-cko^* mouse in its current state is applicable to model KC.

**Figure 6:**
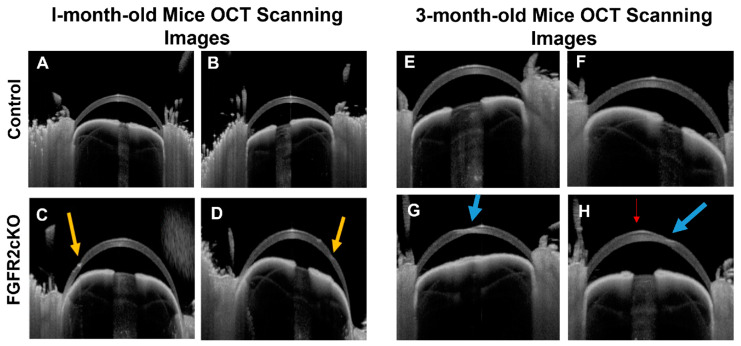
Representative OCT images of corneal thinning in control (**A**,**B**,**E**,**F**) and FGFR2cKO (**C**,**D**,**G**,**H**) mice at 1 month (**A**–**D**) and 3 months (**E**–**H**) old. Arrows indicate thinning (yellow), scarring (blue), and cone-like morphology (red). (Adopted from Joseph et al., https://creativecommons.org/licenses/by/4.0, accessed 29 September 2023) [39].

In the final and most recent publication, by Joseph et al., a stromal keratocyte-specific Fibroblast Growth Factor Receptor 2 (FGFR2) knockout model was described [39]. This model was based on RNA-seq analyses and immunohistochemistry that identified the downregulation of FGFR2 in both normal and KC human corneal fibroblasts [82,83]. FGF signaling is involved in typical eye development by means of cell differentiation, proliferation, migration, and survival [84]. They propose that FGFR2 may be a factor in KC pathogenesis, acting as an initiator of an FGF signaling cascade that results in keratocyte apoptosis [39]. They hypothesize that this model may allow the study of downstream targets of the FGF signaling pathway as well as keratoconus [39]. To examine their model, Joseph et al. utilized immunohistochemistry, Western blotting, terminal deoxynucleotidyl transferase dUTP nick end labeling (TUNEL) assay, slit-lamp biomicroscopy, SD-OCT, corneal topography, and transmission electron microscopy [39]. In the FGFR2 knockout mouse model, they observed progressive, localized stromal thinning consistent with KC at 3 months of age [39]. It is important to note, as seen in Figure 6, that the corneal thinning was not universally localized to the center of the cornea; however, the central thinning seen in Figure 6G is promising as a symptom of KC [39]. Likewise, the corneal angle steepening, changes to the collagen structures, keratocyte apoptosis, and corneal hydrops seen were also analogous to the KC-associated morphological changes seen in humans [39]. It is important to show that the corneal phenotype in this model continues to progress and ultimately develops a gross cone-like morphology. This would likely require aging the mice further to correspond with mid to late adulthood in humans. Despite this, the FGFR2 model demonstrates the development of corneal phenotypes comparable to those in humans. Further, the experimental design used to validate these phenotypes is a thorough assessment of a genetic KC mouse model.

## 3. Rat Models

Rat models are used to a lesser degree than mice in ocular research. One major benefit of using rats instead of mice is the larger corneal diameter while still being relatively easy to house and handle [85]. Rat corneas exhibit a diameter of about 5 mm with an average CCT of 159.08 µm, compared to humans’ 11.7 mm diameter and 0.565 mm CCT (Table 2) [52,56,57,59]. The adult rat cornea is considered fully developed at 8–12 weeks [59]. Like mice and humans, the rat cornea has five layers [60]. Additionally, rats are physiologically similar to humans and have a well-established genome database [85]. A major disadvantage of using rats in a laboratory setting is that they require more space to house, which leads to smaller colony sizes. There are few rat KC models described in the available literature. Furthermore, there are no genetic or spontaneous models. Multiple experiments report inducing some degree of KC in rats using a variety of treatments, including UVR, SAL003, and vitamin A deficiency [40,41,42].

### Treatment-Induced Rat Models

**Figure 7:**
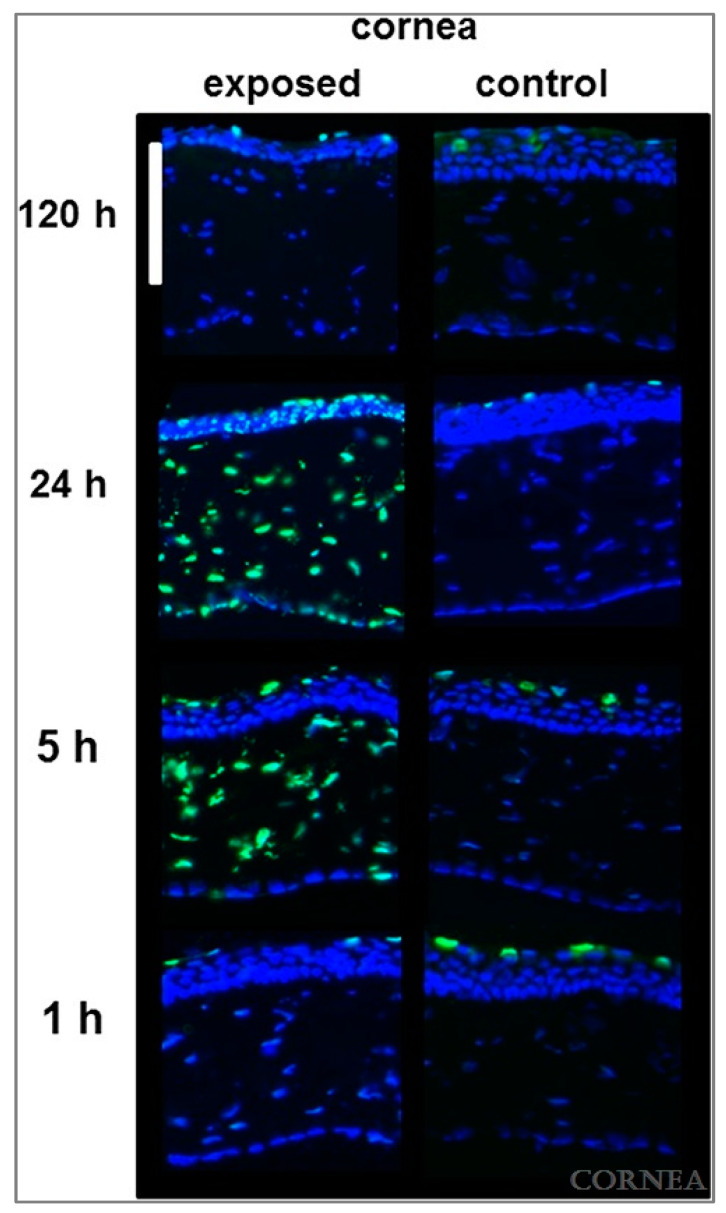
TUNEL staining after in vivo exposure of rat cornea to UVR. Scale bar = 100 μm. (Used with permission of Wolters Kluwer Health, Inc. Philadelphia, PA, USA from Apoptosis in Rat Cornea After In Vivo Exposure to Ultraviolet Radiation at 300 nm, Kronschläger et al., Cornea, Volume 34, Issue 8, p. 945–949, 2015, https://doi.org/10.1097/ico.0000000000000498, accessed 1 November 2023) [40].

Given the previously mentioned relationship between UVR and KC, a model generated by UVR exposure could be a feasible representation of KC. Kronschläger et al. used UVR to induce apoptosis in the epithelium, stroma, and endothelium of Sprague-Dawley rat corneas [40]. Although the study never explicitly mentioned KC or corneal ectasia, their observations were similar to KC histopathology [40]. The authors detailed that, after UVR exposure, each of the corneal layers demonstrated apoptosis (Figure 7) [40]. Importantly, neutrophil infiltration in the stroma was observed [40]. The authors hypothesized that neutrophils may be responsible for the stromal thinning induced by UVR [40]. One major limitation of this study is the failure to quantify the corneal thickness. The study looked primarily at DNA strand breaks via TUNEL assay [40]. This article presents one figure consisting of H&E optical staining, does not provide a scale bar for quantification, and does not detail this in the experimental methods [40]. Despite the limitations in this study, a UVR-induced keratoconus model continues to be a promising option.

To assess the systemic and ocular changes as a result of integrated stress response (ISR) stimulation, Peterson et al. treated rat corneas with an ISR agonist, SAL006 [41]. The integrated stress response is triggered by cellular stress and acts as a cytoprotective pathway, temporarily reducing protein synthesis until cellular stress is resolved [41]. Previously, it was revealed that human keratoconus fibroblasts upregulated ISR compared to human control fibroblasts [86]. After treatment with SAL006, the rat corneas showed reduced stromal keratocyte density, decreased collagenous extracellular matrix production, and the induction of nuclear ATF4 [41]. ATF4 is a transcription factor activated during ISR that regulates the expression of genes targeted by stress induction [87]. Increased activation of this transcription factor in the nucleus indicates that ISR is activated [41]. The study does not mention any morphological changes to the cornea, such as a cone-like shape [41]. The authors do acknowledge that further keratometric assessment is required [41]. Indeed, OCT would be a very beneficial indicator of any localized stromal thinning.

A study by Mutch et al. from 1939 asserted that KC could be induced through vitamin A deficiency in the corneas of rats [42]. Earlier studies identified keratoconus as an unintended result during experiments using vitamin-A-deficient rats [88]. To directly assess keratoconus in vitamin-A-deficient rats, 30 rats were subjected to a vitamin-A-free diet [42]. Some rats suffered from several ocular afflictions, including xerophthalmia, perforation, and keratoconus, which varied in severity [42]. Despite the age of the article, no follow-up studies were done to validate the model. It appears difficult to replicate the results of the experiments due to the inconsistency of the methods. For example, much of the timing of vitamin A administration seemed arbitrary, controls were not mentioned, and animal use protocols seemed absent. Despite the severe KC-like phenotype demonstrated in the article’s lone figure, it is impossible to evaluate the corneas by today’s standards due to the limited technology at the time [42]. It is probable that the description of KC as a characteristic in this study was a misnomer. It would be beneficial to consider H&E staining to evaluate the corneal tissue and OCT to assess central corneal thinning.

## 4. Rabbit Models

The rabbit ocular surface acts as an excellent model for human corneal disorders due to the large eye. In contrast to human corneas, rabbit corneas have a thinner epithelium, a regenerating endothelium, and an absent Bowman’s layer (Table 2) [61,62]. The average CCT in rabbits is 356.11 µm, and the cornea diameter is around 15 mm, by far the largest of the common small mammalian model animals (Table 2) [56,61]. The rabbit CCT reaches its maximum thickness at 18 months [63]. However, the larger size of rabbits in general requires larger cages and, therefore, more housing space and smaller colonies. Similarly to rats, no genetic or spontaneous KC rabbit models have been reported. An interesting similarity between many KC rabbit models is the frequent involvement of collagenase as a treatment to induce a KC phenotype [43,44,45,46,47,48]. These models are often induced with collagenase to study laser in situ keratomileusis (LASIK)-induced corneal ectasia or collagen crosslinking. Collagenase is relevant to KC pathogenesis as it may break down structural collagen fibers in the stroma, thus compromising the structural integrity and thickness of the cornea [72]. Another rabbit model used glucocorticoid treatment to induce corneal ectasia [49,89].

### Treatment-Induced Rabbit Models

A study from Qiao et al. used New Zealand white rabbits to mimic KC corneal ectasia via collagenase type II exposure [43]. Collagenase type II was administered surgically to the rabbits through a corneal trephine and subsequent immersion of the corneas in collagenase type II solution [43]. Rabbit corneas were assessed using slit-lamp biomicroscopy, keratometry (Km), and pachymetry [43]. In vitro assessment consisted of corneal tissue biomechanical stretching and H&E staining [43]. In vivo analysis determined that the experimental group had an increased Km and decreased CCT, indicating a steepened ocular surface and thinning in the center of the cornea [43]. Corneal stress–strain testing proved that the experimental corneas were less stiff [43]. Finally, H&E staining revealed a looser association of collagen fibrils in the stroma [43]. These results are consistent with KC in regard to the abnormal curvature to the eye, central thinning, compromised stiffness, and compromised collagen fibril structural integrity [1]. However, this study failed to produce several important metrics for KC severity and development. To demonstrate that the reduced CCT was localized and that the peripheral thickness did not change, it would have been beneficial to perform OCT for a clearer representation of the phenotype. Additionally, it would have been beneficial to provide post-operative evidence of the rabbit corneas to demonstrate that the model mimicked the gross observations seen in KC patients.

In another study published in 2018, Liu and Yan sought to investigate the role of nuclear related factor 2 (Nrf2) activators in KC pathology [44]. Nrf2 activators like sulforaphane may contribute to an increase in antioxidant gene expression and have a cytoprotective effect against reactive oxygen species in stromal cells [44]. New Zealand white rabbits underwent epithelial debridement and collagenase type II treatment via corneal trephines to induce a KC model [44]. Keratometry revealed a marked increase in the eyes of KC-induced rabbits without any treatments administered [44]. CCT measurements showed a significant decrease in the central thickness of these same KC eyes when compared to normal controls [44]. The H&E stained KC corneas showed very few significant changes after 2 weeks; however, the arrangement of the stromal fibers was much looser in the KC corneas [44]. Further work was done in 2021 by the same group looking to understand the mechanism of corneal degradation in rabbits after collagenase type II treatment [90]. Overall, this study demonstrates that the collagenase induction of KC in rabbits can be employed to assess the pharmacological effects of certain treatments. However, for this method to be fully trusted, an in-depth assessment of the clinical features of the rabbit corneas should be performed. Likewise, it should be noted and considered that these models do not accurately represent any cases of KC in humans, as they do not consider genetic causes and do not exactly mimic the environmental or chemical influences experienced by KC patients as they are not naturally exposed to collagenase.

Japanese white rabbits were used by Kobashi et al. to induce a keratoconus phenotype with collagenase treatment [45]. This procedure consisted of epithelial debridement and collagenase type II treatment via a topical solution, followed by violet light irradiation with riboflavin treatment to treat the KC [45]. This paper was focused mainly on evaluating a new mode of treatment for KC in rabbits. However, it is clear that the collagenase treatment induced a severe cone-like protrusion of the eye [45]. Gross observation of the collagenase-treated eyes showed a very clear, if exaggerated, malformation [45]. Keratometry results revealed that collagenase type II-treated eyes had significant corneal steepening [45]. Likewise, there was significant thinning of the central cornea of the collagenase-treated eyes [45]. This study builds on the hypothesis that collagenase can be used to induce KC in an animal model for downstream studies. For the purpose of this review, it would have been beneficial if Kobashi et al. had performed some additional examinations of the eyes, like slit lamp biomicroscopy, OCT, and histology, to confirm the hallmarks of KC at the tissue level. However, we do recognize that the former two techniques may not be universally available for rabbits.

Cano-Gómez et al. used collagenase type II in New Zealand white rabbits in order to induce KC. The collagenase type II was administered via intrastromal injection [46]. In vivo analysis was performed using keratometry and ex vivo analysis consisted of H&E staining, Sirius Red staining, and RT-PCR [46]. Mean keratometry was increased as a result of collagenase type II treatment, indicating the steepening of the ocular surface [46]. Histology revealed abnormal epithelial arrangement, the loss of collagen fibril organization in Bowman’s layer, and the thickening of the stroma as a result of inflammation [46]. Despite presenting with some of the histological characteristics of KC, this model does not exhibit localized central thinning, stromal thinning, or gross signs of KC. The study notes changes to Bowman’s layer; however, this layer is not present in the rabbit cornea (Table 2) [61,62]. The loss of collagen fiber parallelism and structural integrity identified in their H&E staining as occurring in the Bowman’s layer may actually refer to the stroma, which would be consistent with KC [1,46]. It would be beneficial in the future to evaluate the corneal thickness in these rabbits in vivo, especially in regard to CCT. Likewise, validation of the gross signs of KC would allow for the further assessment of the potential of this model.

A study from Hu et al. determined that collagenase type I treatment was able to degrade the collagen fiber structure and reduce the central thickness in the corneas of New Zealand white rabbits [47]. This study, like others, was used to evaluate the ability of a treatment—in this case, patterned corneal collagen crosslinking—to correct the morphology and mechanical deficits in keratoconic corneas. The method of administration for the collagenase treatment in this study was through exogenous collagenase injection into the stroma [47]. Corneal topography, OCT, and H&E staining were used to confirm the gross clinical phenotype and tissue-level changes to the morphology and structure [47]. The sample size in this study was low, making it difficult to ensure that the phenotype was reliably induced. The dramatic presentation of corneal ectasia in these rabbits is striking but may be more exaggerated than cases of KC in humans.

**Figure 8:**
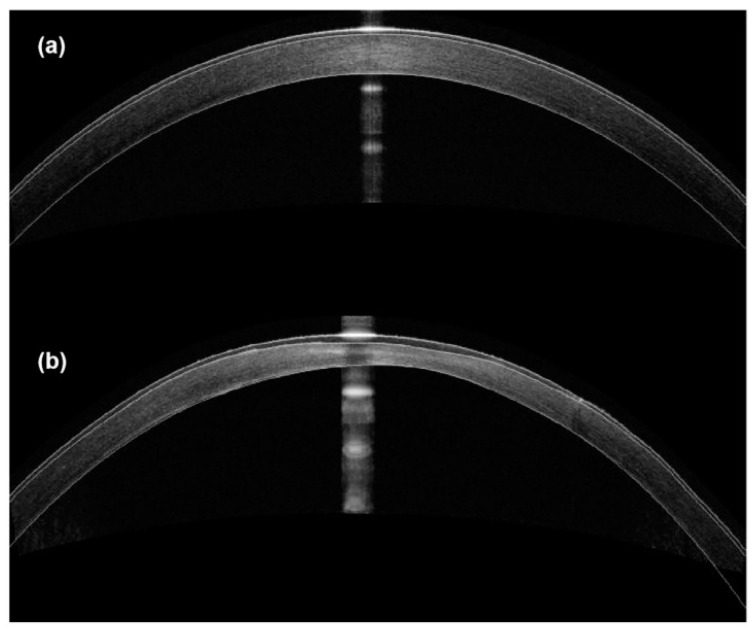
Representative SD-OCT images of rabbit corneas (**a**) before and (**b**) after 4 weeks of collagenase II treatment. (Adopted from Wei et al., https://creativecommons.org/licenses/by/4.0, accessed 29 September 2023) [48].

To induce a model of KC in Japanese white rabbits, Wei et al. also used collagenase type II treatment [48]. This study administered a topical collagenase solution on a cotton pad following epithelial debridement [48]. The focus of the study was to assess the morphology and biomechanical factors in vivo and ex vivo using corneal tomography, spectral domain OCT (SD-OCT), tonometry, stress–strain extensometry, and H&E staining [48]. Collagenase-treated corneas exhibited decreased CCT and lessened biomechanical integrity, which both progressed and were not stable after the 8-week duration of the experiment (Figure 8) [48]. The inability to control the severity of the KC phenotype in this model makes it difficult to use despite the promising pathological developments. However, if a longer-term assessment of this model could be conducted to determine when the model is stable, it could be used to assess KC treatments. 

Corticosteroids have previously been studied in relation to the corneal thickness, loss of stiffness, and loss of biomechanical strength [91]. To study the effects of glucocorticosteroids, generally, on the cornea, Yu et al. utilized a Japanese white rabbit model [49]. The rabbit corneas were treated in vivo with a topical solution of fluorometholone [49]. Ex vivo corneas were measured for CCT, peripheral thickness, and diameter [49]. A mechanical inflation device simulating posterior IOP was used to conduct stress–strain tests to ascertain the biomechanical behavior of the ex vivo rabbit corneas [49]. Material stiffness decreased after an 8-week treatment with fluorometholone as compared to controls [49]. The authors note that the compromised biomechanical integrity caused by corticosteroids may contribute to KC progression, especially regarding post-CXL recurrence [49]. It would be beneficial for this study to be repeated with measurements of the corneal thickness made in live rabbits, as the only in vivo measurement performed was IOP. As this study did not look at KC specifically, and did not set out to characterize any structural phenotypes in the cornea, further studies would be required to fully understand the potential of corticosteroid treatment as a method of inducing KC.

## 5. Proposed Novel Animals

Some additional model animals have been suggested for the study of corneal ectasia but have not yet been employed or established. Avian chicks and tree shrews have been suggested as model organisms to study corneal ectasia. There are certainly benefits to using non-traditional models, especially in focus areas that have experienced difficulties with traditional animal models.

The avian chick eye is closer in corneal diameter to primate than murine eyes (Table 2) [52,65]. The thickness of the chicken cornea measures at around 405 µm; a measure of CCT is not yet established for this model (Table 2) [66]. The chick cornea is composed of 5 layers with a true Bowman’s layer [64]. A 1986 publication from Bitgood and Whitley reported a mutant strain of chick, referred to as pop-eye, with a sex-linked pattern of keratoglobus [87]. Abnormalities in the corneas developed within 5–6 weeks [50]. Some linkage analysis was performed; however, the mutant strain does not appear to have been used beyond this initial report [50,65]. Altered corneal curvature and corneal thinning were also reported with this strain [92]. Despite the assertion that the pop-eye strain of the avian chick presents with hereditary keratoconus, without confirmation of central thinning and histological changes, it is difficult to recognize the pop-eye chick as an appropriate KC model. Furthermore, a later publication from Bitgood refers to the condition as keratoglobus rather than keratoconus, indicating that keratoconus may have been a misnomer for this strain [67].

An emerging model animal in biomedical science is the tree shrew, a small mammal originating from Southeast Asia. One major benefit of the tree shrew is that it is small, weighing about 120–150 g, but it is more closely related to primates than to rodents [68]. The tree shrew cornea measures around 8.5 mm in diameter, which is larger than mouse and rat eyes but smaller than humans (Table 2) [52,69]. Tree shrew corneas have five distinct layers with a similar relative thicknesses to human corneal layers (Table 2) [93]. The CCT of tree shrew corneas ranges from 202 to 301 µm, which is about half of the typical human cornea CCT (Table 2) [57,94]. Crucially, the collagen structure of the Bowman’s layer and stroma is similar in density and arrangement to that of human corneas [93]. Due to their size, it is easier to house and care for tree shrews than primates. However, there are few resources for the genetic modification of tree shrews, and rodents remain easier to house and maintain. Currently, there has been no attempt to generate a KC tree shrew model, and, to the authors’ knowledge, there is no spontaneous KC tree shrew. However, it has been suggested that, due to the structure of collagen fibrils and proteoglycans in the tree shrew, the animal could be a suitable model to study post-LASIK corneal ectasia [93]. Almubrad et al. do specify that the tree shrew cornea could be used to study non-genetic diseases of corneal ectasia; however, it may be possible in the future that the tree shrew could be used for genetic disorders like KC [93].

## 6. Conclusions

Through a review of the currently available literature about keratoconus animal models, we have described and categorized each as a genetic model, spontaneous model, treatment-induced model, or proposed model. The first two groups are limited to mice. All rat and rabbit models discussed, as well as some mouse models, fall into the treated category. The final category is reserved for animals where there have not yet been definitive attempts at creating a KC model; in this review, this includes the tree shrew and avian chick.

The genetic and spontaneous models comprise about one third of the models discussed in this article, with only one spontaneous model. As previously mentioned, these genetic and spontaneous models are only present in studies using mice. Regardless, with the vast number of resources for the genetic modification of mice and the wealth of pre-existing genomic data available, the use of genetic mouse models is reliable and provides many capabilities to researchers. The main factor limiting genetic models is the unclear genetic etiology of KC, which unfortunately may require many failed or incomplete genetic models to fully understand. Herein lies the benefit of a spontaneously occurring KC mouse, as it already has a genetic composition that can cause KC. This genetic background may better mimic the hereditary cases of KC in humans, but likely does not represent the exact genetic makeup of KC in humans.

Treated models may or may not consider the genetic contribution to KC, as genetically modified animals may be administered a treatment. However, of the treated models explored in this article, all were wild-type. Therefore, we can assume that all of the treated models were seeking to represent environmentally induced KC. It is important to note that many of the treated models used the same methods to induce KC. Ultraviolet radiation exposure is used in both mice and rats [26,40]. UVR is likely employed because of its role in breaking down collagen fibers in the cornea, which is antithetical to collagen crosslinking and known as a KC-contributing environmental factor [8,22]. Collagenase is another common method, used in mice and rabbits [28,43,44,45,46,47,48]. Collagenase seems to be especially common in rabbit models, as six of the seven rabbit models discussed utilized collagenase treatments [43,44,45,46,47,48]. It is not clear why collagenase is used more heavily in rabbits than in the other model animals discussed. However, this discrepancy may serve as a potential motivator for collagenase use in mice and rats given the convincing effects of the treatment on rabbit corneas. One interesting possibility for a future study could be a comparison of the effects of UVR or collagenase in mice, rats, and rabbits to assess the techniques as a means of inducing KC in common experimental animals, and this could serve as a guide for studies seeking to induce KC in any of the three species.

Despite the many models discussed, each one has drawbacks that prevent it from being validated. In all of these studies, there is a notable lack of a full, thorough assessment of all the possible variables contributing to the KC pathology. An ideal assessment of a KC model would include the confirmation of clinical features via slit-lamp microscopy; pachymetry confirmed with OCT to assess the central and peripheral thickness, as well as the corneal angle; topography to quantitate and confirm the cone-like protrusion; histology to assess tissue-level changes; immunofluorescence and Western blotting to observe the expression of certain structural proteins; and biomechanical measurements such as stress–strain, IOP, and elastic modulus. Additionally, it would be necessary to use a large sample size, both males and females in a balanced ratio, and animals that have reached maturity in terms of corneal development. However, it is important to note that some of these techniques have not been established in some animals. It is important to note that the use of hand-held pachymetry devices to measure corneal thickness is frequently seen in the studies that we have reviewed; however, hand-held devices only assess the corneal thickness at one point of measurement. OCT is a better option in this regard as it gives a clear representation and quantification of the entire corneal thickness. Since KC is marked by localized thinning of the cornea, it is necessary to measure both the CCT and peripheral corneal thickness to prove that any KC animal model is truly exhibiting KC rather than general corneal ectasia. 

There is clear promise in studies using UVR or collagenase to model KC in animals. However, there is still untapped potential in the genetic and spontaneous KC lines in mice. Genomic studies in these spontaneous lines have and may continue to reveal novel markers that may be associated with heritable incidences of keratoconus [30,31]. Assessing non-coding regions of the SKC/JKC mouse genome may also be a potential route of exploration [30,31]. Due to the complex nature of KC as a genetic and environmentally induced disorder, an interesting possibility would be to mimic both in a mouse model, combining a promising genetic or spontaneous line with an environmental factor such as UVR, eye rubbing, or atopy [10].

The current lack of a validated in vivo model is a critical impediment to the study of KC. Still, several of possible routes may be explored to establish an animal model for KC: the generation of a genetic model based on human KC genomic data; the confirmation of spontaneous animal lines at the tissue, cellular, molecular, and genomic levels; or the treatment of wild-type, genetically modified, or spontaneous KC animals with a combination of chemical or external influences to induce replicable cases of KC that are translational to human cases. Of course, certain methods may be better suited to investigating hereditary cases of KC versus environmentally induced cases. Additional options may become clear as more attempts are made at generating an animal model for KC.

## Figures and Tables

**Table 1:** Literature summaries. Details of the species, strain of given animal, genetic modification, treatments applied, and KC features present in each animal model described here.

Author (Year)	Species	Strain	Genetic Modification	Treatment	KC Features
Newkirk et al. (2007) [26]	Mouse	129S1/SvImJ	None	60% UVA 40% UVB exposure	Cone-like protrusion, loss of keratocytes, stromal thinning, corneal vascularization, corneal fibrosis, keratitis
Ebihara et al. (2008) [27]	Mouse	NC/Nga	None	Eye rubbing/scratching	Cone-like protrusion, epithelial thinning; irregular interface between the epithelium and stroma; epithelial fibrosis; hemidesmosome accumulation in basal cells; deposition of material under epithelial cells; keratocyte deformity; disorganization of stromal collagen fibers; stromal neovascularization
Moghadam et al. (2009) [28]	Mouse	BALB/c	None	Collagenase	Damaged collagen fibrils, epithelial thinning, corneal rupture
Bech et al. (2005) [29]	Mouse	Not specified	Not specified	Photorefractive keratectomy endo-β-galactosidase	Cone-like protrusion, epithelial and stromal thinning, keratocan and β-catenin expression
Tachibana et al. (2002) [30]	Mouse	SKC	None	Castration of males, androgen for females	Cone-like protrusion; large, widely spaced collagen fibrils
Tachibana et al. (2002) [31]	Mouse	Japanese keratoconus (JKC)	None	None	
Quantock et al. (2003) [32]	Mouse	SKC	None	None	
Tost et al. (2005) [33]	Mouse	Not specified	Transgenic murine trisomy 16	None	Corneal hypoplasia, stromal fibrosis, degradation of lens fibers, and loss of compaction in stromal lamellae
Parapuram et al. (2011) [34]	Mouse	*Itgb1 ^f/f^*	Tamoxifen-induced Cre recombinase	None	Stromal thinning, loss of epithelial cell layers, edema, scarring, stromal haze
Stanton et al. (2021) [35]	Mouse	Zfp469 ^BCS/BCS^	CRISPR-Cas9-mediated genome editing	None	Stromal thinning
Khaled et al. (2019) [36]	Mouse	B6N (Cg)-*Ppip5k2^tm1b (EUCOMM)Wtsi^/J*	Gene trap	None	Abnormal corneal surfaces, changes in anterior chamber depth, abnormal corneal curvature, thinning of CCT
Terceiro et al. (2022) [37]	Mouse	C57Bl/6	CRISPR-Cas9-mediated knockout	None	N/A
Wang et al. (2023) [38]	Mouse	*Tgfbr2^kera-cko^*	Conditional knockout	Eye rubbing	Stromal-specific thinning, reduced COL1a1 expression, diminished stromal collagen fibril density
Joseph et al. (2023) [39]	Mouse	FGFR2 KO	Inducible keratocyte-specific Cre mice	None	Localized stromal thinning
Kronschläger et al. (2015) [40]	Rat	Sprague-Dawley	None	UVR	Apoptosis in all corneal layers and neutrophil infiltration in the stroma
Peterson et al. (2021) [41]	Rat	Sprague-Dawley	None	Topical SAL003 nanosuspension	Decreased keratocyte density and reduced Col1A1 transcripts
Mutch et al. (1939) [42]	Rat	Not specified	None	Low vitamin A diet	Cone-like protrusion
Qiao et al. (2018) [43]	Rabbit	New Zealand White	None	Collagenase type II, epithelial debridement	Steepened ocular surface, central thinning, loss of corneal stiffness, loose association of stromal collagen fibrils
Liu and Yan (2018) [44]	Rabbit	New Zealand White	None	Collagenase type II, zinc (II) protoporphryin IX, sulforaphane	Increased corneal steepness, central thinning, loose stromal fiber association
Kobashi et al. (2023) [45]	Rabbit	Japanese White	None	Violet light, collagenase type II	Cone-like protrusion, increased corneal steepness, central thinning
Cano-Gomez et al. (2023) [46]	Rabbit	New Zealand White	None	Collagenase type II intrastromal injection	Increased corneal steepness, abnormal epithelial arrangement, loss of collagen fibril arrangement, inflamed stroma
Hu et al. (2021) [47]	Rabbit	New Zealand White	None	Intrastromal collagenase type I injection	Central thinning and degraded collagen fiber structure
Wei et al. (2023) [48]	Rabbit	Japanese White	None	Intrastromal collagenase type I injection	Progressive central thinning and compromised biomechanical integrity
Yu et al. (2014) [49]	Rabbit	Japanese White	None	Topical fluorometholone	Decreased biomechanical stiffness
Bitgood and Whitley (1986) [50]	Avian chick	Pop-eye (*pop*)	None	None	Keratoglobus and increased anterior chamber depth

**Table 2:** Comparison of KC model organisms. Comparison of cornea diameter, central corneal thickness, corneal layers, and age of corneal maturation between mouse, rat, rabbit, avian chick, tree shrew, and human.

Species	Cornea Diameter (mm)	Average CCT (μm)	Corneal Layers	Age at Corneal Maturation	References
Mouse	2.3–2.6	106.0	4–5 layers (strain-dependent, some lack Bowman’s layer)	8 weeks	Henricksson et al. [51], Schulz et al. [56], Hanlon et al. [58], Smith et al. [53], Wilson [54]
Rat	5	159.08	5 layers	8–12 weeks	Schulz et al. [56], He et al. [59], Hayashi et al. [60]
Rabbit	15	356.11	4 layers, lacking Bowman’s layer	18 months	Schulz et al. [56], Peiffer at al. [61], Wilson et al. [62], Zhang et al. [63]
Avian Chick	9.1	405 (overall corneal thickness)	5 layers	n/a	Ritchey et al. [64], Fowler et al. [65], Wisely et al. [66]
Tree Shrew	8.5	202–301	5 layers	n/a	Jasien et al. [67], Almubrad et al. [68], Wu et al. [69]
Human	11.7	565.0	5 layers	~20 years	Rüfer et al. [52], Doughty et al. [57], Knox Cartwright et al. [70]

## Data Availability

Not applicable.
